# Economic Burden of Endoscopic Vacuum Therapy Compared to Alternative Therapy Methods in Patients with Anastomotic Leakage After Esophagectomy

**DOI:** 10.1007/s11605-021-04955-w

**Published:** 2021-02-24

**Authors:** Ann-Kathrin Eichelmann, Sarah Ismail, Jennifer Merten, Patrycja Slepecka, Daniel Palmes, Mike G. Laukötter, Andreas Pascher, Wolf Arif Mardin

**Affiliations:** 1 Department of General, Visceral and Transplant Surgery, University Hospital of Muenster, Albert-Schweitzer-Campus 1, W1, 48149 Muenster, Germany; 2 Department of Medical Controlling, University Hospital of Muenster, Nils-Stensen-Str. 8, 48149 Muenster, Germany

**Keywords:** Esophageal cancer, Anastomotic leak, Endoscopic vacuum therapy, Stent, Profit center analysis

## Abstract

**Background:**

Endoscopic vacuum therapy (EVT) has become a promising option in the management of anastomotic leakage (AL) after esophagectomy. However, EVT is an effortful approach associated with multiple interventions. In this study, we conduct a comparative cost analysis for methods of management of AL.

**Methods:**

All patients who experienced AL treated by EVT, stent, or reoperation following Ivor Lewis esophagectomy for esophageal cancer were included. Cases that were managed by more than one modality were excluded. For the remaining cases, in-patient treatment cost was collected for material, personnel, (par)enteral nutrition, intensive care, operating room, and imaging.

**Results:**

42 patients were treated as follows: EVT *n* = 25, stent *n* = 13, and reoperation *n* = 4. The mean duration of therapy as well as length of overall hospital stay was significantly shorter in the stent than the EVT group (30 vs. 44d, *p* = 0.046; 34 vs. 53d, *p* = 0.02). The total mean cost for stent was €33.685, and the total cost for EVT was €46.136, resulting in a delta increase of 37% for EVT vs. stent cost. 75% (€34.320, EVT), respectively, 80% (€26.900, stent) of total costs were caused by ICU stay. Mean pure costs for endoscopic management were relatively low and comparable between both groups (EVT: €1.900, stent: €1.100, *p* = 0.28).

**Conclusion:**

Management of AL represents an effortful approach that results in high overall costs. The expenses directly related to EVT and stent therapy were however comparatively low with more than 75% of costs being attributable to the ICU stay. Reduction of ICU care should be a central part of cost reduction strategies.

## Introduction

Anastomotic leakage (AL) represents a major complication following esophagectomy, occurring in about 10% of cases—even in high-volume centers.^1^, ^2^ Some individual studies even report leakage rates of up to 49%.^3^ In recent years, endoscopic management via stent placement or endoscopic vacuum therapy (EVT) has become the standard of care in the treatment of such leakages, drastically reducing the need for reoperation.^4^ Insertion of a self-expanding metal stent (SEMS) was the main endoscopic approach to treat esophageal leakages with clinical success rates of up to 87%,^5^, ^6^ but EVT has become ever more available. Studies comparing these treatment modalities are rare, and those that do exist feature only small patient numbers. However, there is budding evidence that suggests that EVT might be superior to stent therapy.^7^–^11^ Advantages of EVT over SEMS are reduced bacterial contamination, secretion and local edema, and promotion of granulation and perfusion.^4^ These benefits come at a cost as EVT is a labor-intensive approach: the sponge needs to be changed twice a week—requiring general anesthesia in some cases—and therapy might be required over the course of several weeks. A high financial burden is the likely outcome of EVT, comprised of personnel costs, increased intensive care unit (ICU) stay, increased overall inpatient stay, and necessity of parenteral nutrition as well as control imaging.

It is well known that complications following esophageal surgery result in a substantial increase in costs—AL, for example, was associated with a cost increase of €4.123 per case.^12^, ^13^ Further, Baltin et al. were able to quantify an inverse correlation between severity of complications according to Clavien-Dindo and profit margins (e.g., I: €-2.878, IVb: €-58.543). In their study, only patients suffering from no complications generated a marginally positive profit margin of €2.514.^13^

The reduction of costs is an omnipresent challenge in the current medical landscape with ever-increasing economic pressure. Many study groups have examined the effectiveness of EVT and investigated different indications,^14^–^16^ but data on economic ramifications is sparse. Therefore, the goal of this study was to quantify and compare costs incurred by the main complication management methods for AL after esophageal resection.

## Methods

### Case Selection

Patients with AL following Ivor Lewis esophagectomy for esophageal cancer were identified between the years 2009 and 2015. Of these, cases managed by more than one treatment modality, i.e., change of treatment, were excluded for further analysis. This also applied to patients who underwent reoperation after initial conservative management of anastomotic leakage. However, patients who underwent reoperation because of other reasons than AL (e.g., wound dehiscence after laparotomy and implementation of a feeding tube) were included. The reasoning behind this approach was to display the incurred costs for each treatment modality separately. The primary endpoint was successful leak closure.

All patients underwent esophageal resection with intrathoracic anastomosis (Ivor Lewis procedure). Peri- and postoperative management was performed as described previously.^17^, ^18^ If AL was suspected, an immediate endoscopy with application of a contrast agent as well as a CT scan was performed.

The study has been approved by the institutional review committee (2018-208-f-S), and it conforms to the provisions of the Declaration of Helsinki. Informed consent was obtained from all individual participants included in the study.

### Study Parameters

Besides demographics and tumor characteristics, the following parameters were analyzed: day of occurrence of leakage, type and duration of intervention (stent, EVT, and reoperation), hospital/ICU stay, in-hospital mortality, necessity and duration of total parenteral nutrition (TPN), necessity of central venous catheter, and radiologic imaging. In this regard, emphasis was put on the financial aspects of the complication management therapy, especially of EVT vs. stent treatment. Costs were collected from the controlling department of the hospital.

The selection of the treatment modality was at the discretion of the responsible surgeon. The approach at our institution changed in 2012 with the introduction of EVT therapy. Before 2012, all endoscopically treated patients received stents; starting in 2012, EVT was the first-line strategy for endoscopic management.

### Conservative Complication Management: Stent or EVT

Depending on the general condition of the patient, endoscopic interventions were performed either under sedation (with midazolam and propofol) or under general anesthesia. Costs for personnel and material were collected, and the average cost per intervention was calculated.

### Stent Placement

Endoscopic stent placement was performed as described previously.^7^ Briefly, after endoscopic lavage of the mediastinal leakage cavity, a partially covered 10 cm long (7 cm covered) self-expanding nitinol stent (Ultraflex®, Boston Scientific Corp., USA) or a fully covered self-expanding nitinol stent (aixstent®, Leufen Medical GmbH, Germany) was placed over the leakage. For details of the stents, see also.^7^ Reendoscopy after stent insertion and X-ray contrast study were used to ascertain correct stent positioning and sealing. Additional endoscopic interventions were only performed on demand. Stent removal was performed after a period of 4 to 6 weeks.

### Endoscopic Vacuum Therapy

EVT has replaced stent therapy as a gold standard in our institution since 2012 and was performed as described previously.^4^, ^7^ Our surgical endoscopic unit does not use commercially available sponge sets but a polyurethane sponge (VivanoMed Foam, Hartmann AG) that is cut to the required dimensions and then either placed endoluminally or into the defect cavity. Continuous negative pressure of 100-125 mmHg was applied (VivanoTec, Hartmann AG) via a polyvinyl chloride gastroduodenal tube. Sponges were changed every 3 (intracavitary) or 5 (endoluminal) days. Therapy was defined as successful in the case of (1) defect cavity lined with surface epithelium and (2) contrast swallow study without detection of leakage.

### Reoperation

Patients who did not qualify for conservative treatment (e.g., too large defect and progressive necrosis) underwent reoperation as first-line therapy. This was carried out via oversewing of the anastomosis, reanastomosis, or discontinuity resection.

### Nutrition

During complication management, nutrition was ensured either via TPN, parenteral feeding, a transnasal enteral, or a jejunostomy feeding tube. In the case of stent implementation, oral intake was allowed when sealing of the stent was complete prior to which patients received TPN. In the case of EVT, patients were allowed oral intake of fluids with the sponge placed intracavitary. However, this study population as well as in the case of endoluminal EVT received TPN for the duration of EVT therapy. Following successful EVT including removal of the sponge, oral feeding was commenced starting with 400 ml fluids per day. Costs were collected for the duration of therapy, including insertion of numerous central venous catheters in the case of TPN.

### Statistics

Data were expressed as numbers with percentages or as mean/median with range. Statistics were performed using nonparametric tests (Mann-Whitney test), one-way ANOVA for multiple groups and a Fisher’s exact test for categorical variables. A *p* value <0.05 was considered significant. Statistical analysis was performed using SPSS 26 (IBM Corp., USA) and PRISM 8 for macOS (GraphPad Software 2019).

## Results

64 patients with AL were identified over the study period. 22 patients were excluded due to a switch in treatment modality (*n* = 14) and/or death prior to successful leak closure (*n* = 9), resulting in the following numbers for the subgroups: EVT *n* = 25, stent *n* = 13, and *n* = 4 surgery (*n* = 1 oversewing of the anastomosis, *n* = 3 discontinuity resection). See Table 1 for demographic data. Groups were comparable in terms of demographic data and staging investigations. 74% of the patients received neoadjuvant (radio)chemotherapy.

**Table 1:** Study population/demographics (*n* = 42)

	EVT *n* = 25	Stent *n* = 13	Surgery *n* = 4	*p*
Sex				
→Male	22 (88%)	10 (77%)	4 (100%)	0.45
→Female	3 (12%)	3 (23%)	0	
Age (years)				
→Mean	60.4	60.2	69.0	0.24
→Median	60 (42-78)	65 (37-88)	68 (65-76)	
Type of tumor				
→EAC	20 (80%)	10 (77%)	2 (50%)	0.54
→SCC	4 (16%)	2 (15%)	2 (50%)	
→Others	1 (4%)	1 (8%)	0	
Preoperative staging				
→uT1	1 (4%)	1 (8%)	0	0.86
→uT2	6 (24%)	1 (8%)	1 (25%)	
→uT3	15 (60%)	9 (70%)	3 (75%)	
→uT4	1 (4%)	0	0	
→N positive	19 (76%)	8 (62%)	3 (75%)	0.79
→N negative	4 (16%)	3 (23%)	1 (25%)	
→Missing	2 (8%)	2 (15%)	0	
Neoadjuvant therapy				
→Yes	20 (80%)	8 (62%)	3 (75%)	0.2
→CTx	6	4	0	
→RCTx	14	4	3	
Intention				
→Curative	25 (100%)	10 (77%)	4 (100%)	0.03
→Palliative	0	3 (23%)	0	
Postoperative staging				
→ (y)pT0	2 (8%)	2 (15%)	2 (50%)	0.54
→ (y)pT1	6 (24%)	2 (15%)	0	
→ (y)pT2	3 (12%)	2 (15%)	1 (25%)	
→ (y)pT3	12 (48%)	7 (54%)	1 (25%)	
→ (y)pT4	1 (4%)	0	0	
→N0	11 (44%)	8 (62%)	3 (75%)	0.53
→N1	6 (24%)	4 (31%)	0	
→N2	4 (16%)	0	1 (25%)	
→N3	3 (12%)	1 (8%)	0	
Removed lymph nodes (median)	25 (11-39)	19 (12-42)	18 (10-34)	0.09
Positive lymph nodes (mean)	2.7	1.2	2.3	0.33
→G1	1 (4%)	2 (15%)	2 (50%)	0.2
→G2	6 (24%)	2 (15%)	1 (25%)	
→G3	9 (36%)	8 (62%)	1 (25%)	
→R0	21 (84%)	11 (85%)	3 (75%)	0.44
→R1	3 (12%)	2 (15%)	1 (25%)	
Overall survival (years, mean)	2.91	3.54	4.63	0.7

*EAC* adenocarcinoma, *SCC* squamous cell carcinoma, *CTx* chemotherapy, *RCTx* radiochemotherapy

### Complication Management: EVT vs. Stent

The mean time to diagnosis of AL was 10 days following esophagectomy in both groups. In the EVT group, the mean duration of therapy was 30 days, and sponges were changed 7.4 times (mean) with 4.6 endoscopic procedures performed under general anesthesia and 2.8 under sedation (Table 2).

**Table 2:** Management of anastomotic leakage: EVT vs. stent

	EVT *n* = 25	Stent *n* = 13	*p*
Interval until initiation of EVT/stent following surgery (d)	9.5 (1–41)	9.5 (3–17)	0.4
Duration of therapy (d)			
Mean	30 ± 25	44.4 ± 20	0.046
Median	23 (3–101)	44 (11–68)	
Number of EVT/stents per patient	7.4 (1–25)	1.5 (1–3)	
→Placement under			
- Sedation	2.8 (0–8)	0.7 (0–3)	
- General anesthesia	4.6 (0–20)	0.8 (0–2)	
Nutrition mode			
→Parenteral nutrition	19 (76%)	13 (100%)	
→Transnasal enteral feeding tube	4 (16%)	4 (31%)	
→J-tube	6 (24%)	0	
Length of TPN (d)	35.8 (9–107)	22.7 (10–48)	0.056
Number of central venous catheters	2.4 (0–10)	0.54 (0–3)	0.002
Length of overall stay (d)			0.02
Mean	53	34	
Median	47 (14–119)	34 (17–56)	
Length of total ICU stay (d)			
Mean	26	21	0.68
Median	18 (4–107)	18 (8–38)	
→Intensive care			
→Mean	9	7	0.36
→Median	4 (1–37)	2 (1–26)	
→Intermediate care			
→Mean	17	13	0.9
→Median	12 (2–78)	11 (3–26)	
Number of chest X-rays	9.2 (0–32)	12.2 (6–21)	0.05
Number of CT-scans (thorax/abdomen)	1.8 (0–6)	1.8 (0–4)	0.86
Number of gastrografin swallows	0.32 (0–2)	1.6 (0–4)	0.0001

*d* days, *TPN* total parenteral nutrition

In the stent group, the mean duration of therapy was 44 days; the stent was changed 1.5 times (6 of 13 patients needed more than one stent) with 0.8 endoscopic procedures performed under general anesthesia and 0.7 under sedation. The length of overall hospital stay was significantly shorter in the stent group as patients could be discharged after successful stent implantation and establishment of oral food intake (34 vs. 53 days, *p* = 0.02). Patients stayed approximately half of their overall stay in ICU (EVT 26 days, stent 21 days, *p* = 0.68).

The majority of patients required TPN via a central catheter; only few patients received nutrition via a transnasal enteral feeding tube or a J-tube. Oral food intake was achievable earlier in the stent group vs. the EVT group leading to reduced duration of TPN for patients in the stent group (23 vs. 36 days, *p* = 0.056) and fewer changes of central catheters (0.5 vs. 2.4, *p* = 0.002, Table 2).

Regarding imaging over the postoperative course, patients in both groups received an average of 2 CT-scans. However, X-rays and gastrografin swallow studies were more often performed in the stent group (Table 2).

### Costs: EVT vs. Stent

Complication management via EVT resulted in total costs of €46.000 per case vs. €34.000 per case for treatment via stent (*p* = 0.19, Table 3). The majority of costs were related to ICU treatment (EVT: €34.000 = 75% of total costs, stent: €27.000 = 80% of total costs). Mean costs for endoscopic management were relatively low (EVT: €1.900, stent: €1.100, *p* = 0.28). The highest proportion of intervention costs in the stent group was caused by the high costs of the stent itself (mean €690).

**Table 3:** Incurred costs

	EVT *n* = 25	Stent *n* = 13	Surgery *n* = 4	*p*
Costs endoscopy (€)				0.28
Mean	1.940 ± 2006	1.062 ± 456	–	
Median	1401 (218-7.343)	690 (690-2.070)		
Number of performed reoperations			2.5 (1–4)	<0.0001^*^
Costs surgery (€)	–	–		
Mean			7.910 ± 3.914	
Median			7.910 (4.520–11.300)	
Costs normal ward (€)				0.03
Mean	8.432 ± 5857	4.197 ± 2453	4.418 ± 3371	
Median	7.750 (1.550–27.900)	4.340 (0–8.060)	3.875 (930–8.990)	
Costs intensive care (€)				0.06
Mean	34.320 ± 29.928	26.900 ± 14.015	64.350 ± 34.971	
Median	23.400 (5.200–139.100)	23.400 (10.400–49.400)	75.400 (15.600–91.000)	
Costs nutrition (€)				0.6
Mean	524 ± 404	415 ± 234	411 ± 226	
Median	462 (109–2.090)	418 (213–1.056)	402 (154–688)	
Costs central venous catheters (€)				<0.001
Mean	193 ± 118	32 ± 53	30 ± 35	
Median	180 (60–600)	0 (0–180)	30 (0–60)	
Costs radiology (€)				0.02
Mean	774 ± 647	1.080 ± 519	1.748 ± 1039	
Median	629 (68–2.890)	1.094 (404–1.881)	1.978 (427–2608)	
Total costs (€)				0.03
Mean	46.136 ± 31.759	33.685 ± 14.809	78.867 ± 42.282	
Median	35.172 (12.243–155.743)	25.517 (17.040–55.801)	91.001 (21.691–111.773)	
Total costs without intensive care (€)				0.03
Mean	11.816 ± 7037	6.785 ± 3040	14.517 ± 8.010	
Median	11.069 (3.243–36.542)	6.401 (2.117–12.458)	14.301 (6.091–23.373)	

^*^Compared to costs for endoscopy

### Reoperation

Only *n* = 4 patients underwent reoperation as first-line therapy (*n* = 1 oversewing of the anastomosis, *n* = 3 discontinuity resection). Overall postoperative costs were significantly higher with €78.870 per case (*p* = 0.03). The mean ICU stay of 50 days (± 27 days) was prolonged vs. the other groups (*p* = 0.06), resulting in ICU costs of €64.350. Regarding the costs after exclusion of ICU stay, these were €14.520 still significantly higher than expenses that occurred in the endoscopic management of complications (*p* = 0.03). As a major cost factor, the significantly higher mean costs of €7.910 of the reoperation itself compared to costs of €1.940 (EVT), respectively, 1.060€ (stent, *p* < 0.0001) have contributed to these high expenses.

## Discussion

Despite improvements in surgical technique, such as fully robotic abdominothoracic esophagectomy, AL after esophagectomy remains a significant complication. While reoperations and the implementation of stents were the first-line therapy in the past, management of AL has been revolutionized by EVT. Data from our own group showed that EVT might be superior to stent placement in the management of this important complication.^7^ There is abundant and growing literature on the subject of appropriate treatment of anastomotic leaks—the objective of this study was altogether different as we sought to investigate the economic ramifications of complication management. The necessity of multiple interventions, high personnel costs, and increased duration of in-patient care suggests that effective therapy results in high costs which surpass revenue. With increasing economic pressure, cost reduction strategies are of great interest. Therefore, our aim was to quantify and compare cost differences between treatment approaches for anastomotic leakage. While it was not unexpected that EVT was associated with higher costs than stent placement, we show detailed cost analysis that can be the basis for cost reduction strategies. We decided to include the costs associated with primary reoperation to paint a more complete picture of the management pathways. However, this patient group represents a different and far greater clinical challenge and should not be directly compared to the stent or EVT groups. It must also be noted that costs incurred—even the very definition of costs incurred—are a nonstandardized measure. Our numbers reflect the situation at our institution, and there currently exists no calibration reference with which to normalize our data in regard to other hospitals. There will be significant interhospital variations both on the physical level of resources used and on the controlling level of resource cost to case attribution.

The total mean cost for stent was €33.685, and the total cost for EVT was €46.136. This shows a delta of €12.451 which amounts to an increase of 37% for EVT vs. stent cost. The largest cost factor in both groups was by far the ICU stay. This factor is in general not significantly influenced by the treatment algorithm but primarily dependent on the severity of the complications. Thus, the manageable cost difference was examined, defined as all costs except ICU stay. Here, we arrive at smaller absolute numbers (€6.785 for stent; €11.816 for EVT) but an even higher relative cost increase of 74% (delta of €5.301) for EVT vs. stent. The remaining major cost factor after exclusion of ICU stay was the cost for the stay at the normal ward. The length of this stay is more closely associated with the treatment method than complication severity as EVT therapy requires a prolonged stay due to multiple necessary interventions.

A few studies have addressed the economic burden arising from complications after esophagectomy.^12^, ^13^, ^19^, ^20^ All of these only recently published studies conclude that complications are associated with a substantial increase in costs. While Goense et al. observed a per case cost increase of €4.123 for AL,^12^ the median standardized costs per leak described by Agzarian et al. were significantly higher with $68.296. Reported mean costs were even higher at $119,822. The authors attributed the 2.6 greater mean treatment costs mainly to prolonged hospitalization, and—in line with our findings—to length of ICU stay. Further, Agzarian et al. report a treatment time for AL of 73 days, while the median duration in our study was 23 (EVT) and 44 (stent) days.^20^

Another group showed surgical treatment even in high-volume centers to be nonprofitable, i.e., costs surpassing DRG (Diagnosis Related Groups)-revenue, with a mean profit margin of €-1.747. In their analysis, only completely complication-free cases following transthoracic esophagectomy generated a positive profit margin of €2.514, while the case of a Clavien-Dindo IVb complication deficit was €-58.543.^13^ These numbers impressively demonstrate the importance of further minimizing complications, for reasons of patient safety but also from an economical point of view.

Beyond these studies that analyzed the increased costs because of complications following esophagectomy, there is to the best of our knowledge only one study available that performed a cost analysis for the endoscopic management of AL by comparing costs for EVT vs. stent.^21^ The authors calculated from their InEK (Institute for Remuneration System in Hospitals) analysis of *n* = 21 patients almost twice the deficit for EVT compared to stent treatment (EVT: €-9.282, stent: €-5.156 per average case). The higher costs were explained by higher personnel and material costs as well as a prolonged hospital stay in case of EVT. Moreover, the deficit in the case of EVT can also be attributed to the fact that in contrast to stent therapy, costs for EVT are not directly compensated and the authors plead for initiation of financial reimbursement for EVT. For example, introducing extra fees for direct cost coverage or new coding conditions in the DRG system could contribute to a comparable economic outcome in the EVT cohort.^21^ Their results are insofar in line with our observations that EVT was associated with higher costs via a prolonged hospital stay compared to stent treatment.

There are some limitations of the current study that need consideration. One potential drawback of this study is that our cases stem from the early years of EVT therapy during which algorithms were still being refined (EVT has replaced stent therapy as a gold standard in our institution since 2012, resulting in EVT as first-line therapy in the management of AL and the fact that no further patients received stent therapy as first choice as of this date). This may have been the cause for a higher number of overall endoscopies and especially a higher number of interventions under general anesthesia. Current developments may lead to more efficient and less time-consuming EVT strategies, thus severely shrinking the cost increase. This is however not yet reflected in the literature with Baltin et al. reporting an average length of stay of 29.44 days for stent treatment and 35.23 days for EVT.^21^ This study, much like ours, suffers from a small case load, limiting its statistical power. The small case load of only *n* = 4 must especially be considered when interpreting the results of the costs that occurred for the study population who underwent reoperation. Secondly, these patients suffered from larger defects which did not qualify for endoscopic management. This selection bias may also be responsible for the significant prolonged hospital stay and increased costs. However, these small numbers reflect the pleasant fact that only a very small percentage of patients undergo a reoperation as first-line therapy, even in the study period of 2009–2015, and secondly, after exclusion of ICU stay, costs were still significantly higher than expenses that occurred in the conservative management of complications. As a final limitation, we need to mention that our endoscopy unit uses individually prepared sponges. This represents a cost-effective solution compared to the commercially available sponges since one foam can be cut to 4–5 smaller foams used for EVT. However, this approach may not reflect standards used elsewhere (as, e.g., demonstrated in^22^).

## Conclusion

Despite these shortcomings, it remains clear that management of this severe complication of esophagectomy still presents a cost and labor-intensive process. The majority of costs that occur in the complication management are attributable to the ICU length of stay. While the pure costs for the endoscopic management of €1.900 for EVT were higher than the costs for stent therapy (€1.100), this difference did not reach statistical significance, and the EVT expenses were—considering the high overall costs of complication management—comparatively low. Therefore, both treatment modalities are justifiable from an economic point of view. Cost reduction strategies should primarily focus on possibilities to decrease the length of ICU stay since the latter represented by far the largest cost factor in both groups.

## References

[1] Messager M, Warlaumont M, Renaud F, Marin H, Branche J, Piessen G, Mariette C (2017). Recent improvements in the management of esophageal anastomotic leak after surgery for cancer. Eur J Surg Oncol.

[2] Markar S, Gronnier C, Duhamel A, Bigourdan JM, Badic B, du Rieu MC, Lefevre JH, Turner K, Luc G, Mariette C (2015). Pattern of postoperative mortality after esophageal cancer resection according to center volume: results from a large European multicenter study. Ann Surg Oncol.

[3] Kamarajah, S.K., Lin, A., Tharmaraja, T., Bharwada, Y., Bundred, J.R., Nepogodiev, D., Evans, R.P.T., Singh, P., Griffiths, E.A.: Risk factors and outcomes associated with anastomotic leaks following esophagectomy: a systematic review and meta-analysis. Dis Esophagus 33(3) (2020). doi:10.1093/dote/doz089 10.1093/dote/doz089 31957798

[4] Laukoetter MG, Mennigen R, Neumann PA, Dhayat S, Horst G, Palmes D, Senninger N, Vowinkel T (2017). Successful closure of defects in the upper gastrointestinal tract by endoscopic vacuum therapy (EVT): a prospective cohort study. Surg Endosc.

[5] Dasari BV, Neely D, Kennedy A, Spence G, Rice P, Mackle E, Epanomeritakis E (2014). The role of esophageal stents in the management of esophageal anastomotic leaks and benign esophageal perforations. Annals of surgery.

[6] Kamarajah, S.K., Bundred, J., Spence, G., Kennedy, A., Dasari, B.V.M., Griffiths, E.A.: Critical appraisal of the impact of oesophageal stents in the management of oesophageal anastomotic leaks and benign oesophageal perforations: an updated systematic review. World J Surg (2019). doi:10.1007/s00268-019-05259-6 10.1007/s00268-019-05259-6 31686158

[7] Mennigen R, Harting C, Lindner K, Vowinkel T, Rijcken E, Palmes D, Senninger N, Laukoetter MG (2015). Comparison of endoscopic vacuum therapy versus stent for anastomotic leak after esophagectomy. J Gastrointest Surg.

[8] Schniewind B, Schafmayer C, Voehrs G, Egberts J, von Schoenfels W, Rose T, Kurdow R, Arlt A, Ellrichmann M, Jürgensen C, Schreiber S, Becker T, Hampe J (2013). Endoscopic endoluminal vacuum therapy is superior to other regimens in managing anastomotic leakage after esophagectomy: a comparative retrospective study. Surg Endosc.

[9] Brangewitz M, Voigtländer T, Helfritz FA, Lankisch TO, Winkler M, Klempnauer J, Manns MP, Schneider AS, Wedemeyer J (2013). Endoscopic closure of esophageal intrathoracic leaks: stent versus endoscopic vacuum-assisted closure, a retrospective analysis. Endoscopy.

[10] Palmes, D., Kebschull, L., Bahde, R., Senninger, N., Pascher, A., Laukötter, M.G., Eichelmann, A.K.: Management of nonmalignant tracheo- and bronchoesophageal fistula after esophagectomy. Thorac Cardiovasc Surg (2020). doi:10.1055/s-0039-1700970 10.1055/s-0039-1700970 32114691

[11] Rausa, E., Asti, E., Aiolfi, A., Bianco, F., Bonitta, G., Bonavina, L.: Comparison of endoscopic vacuum therapy versus endoscopic stenting for esophageal leaks: systematic review and meta-analysis. Dis Esophagus 31(11) (2018). doi:10.1093/dote/doy060 10.1093/dote/doy060 29939229

[12] Goense L, van Dijk WA, Govaert JA, van Rossum PS, Ruurda JP, van Hillegersberg R (2017). Hospital costs of complications after esophagectomy for cancer. Eur J Surg Oncol.

[13] Baltin, C.T., Bludau, M., Kron, F., Zander, T., Hallek, M., Hölscher, A.H., Schröder, W.: [Profit center analysis of esophagectomy : economical analysis of transthoracic esophagectomy depending on postoperative complications]. Chirurg 89(3), 229-236 (2018). doi:10.1007/s00104-018-0590-9 10.1007/s00104-018-0590-9 29417163

[14] Jung, C.F.M., Müller-Dornieden, A., Gaedcke, J., Kunsch, S., Gromski, M.A., Biggemann, L., Seif Amir Hosseini, A., Ghadimi, M., Ellenrieder, V., Wedi, E.: Impact of endoscopic vacuum therapy with low negative pressure for esophageal perforations and postoperative anastomotic esophageal leaks. Digestion, 1-11 (2020). doi:10.1159/000506101 10.1159/000506101 32045916

[15] Loske G (2019). Endoscopic negative pressure therapy of the upper gastrointestinal tract. Chirurg.

[16] de Moura DTH, de Moura BFBH, Manfredi MA, Hathorn KE, Bazarbashi AN, Ribeiro IB, de Moura EGH, Thompson CC (2019). Role of endoscopic vacuum therapy in the management of gastrointestinal transmural defects. World J Gastrointest Endosc.

[17] Lindner, K., Lübbe, L., Müller, A.K., Palmes, D., Senninger, N., Hummel, R.: Potential risk factors and outcomes of fistulas between the upper intestinal tract and the airway following Ivor-Lewis esophagectomy. Diseases of the esophagus : official journal of the International Society for Diseases of the Esophagus / I.S.D.E 30(3), 1-8 (2017). doi:10.1111/dote.12459 10.1111/dote.12459 27060908

[18] Muller, A.K., Lenschow, C., Palmes, D., Senninger, N., Hummel, R., Lindner, K.: [Timing of esophagectomy in multimodal therapy of esophageal cancer : impact of time interval between neoadjuvant therapy and surgery on outcome and response.]. Chirurg (2015). doi:10.1007/s00104-014-2916-6 10.1007/s00104-014-2916-6 25662991

[19] Guo, L.W., Shi, C.L., Huang, H.Y., Wang, L., Yue, X.P., Liu, S.Z., Li, J., Su, K., Dai, M., Sun, X.B., Shi, J.F.: [Economic burden of esophageal cancer in China from 1996 to 2015: a systematic review]. Zhonghua Liu Xing Bing Xue Za Zhi 38(1), 102-109 (2017). doi:10.3760/cma.j.issn.0254-6450.2017.01.020 10.3760/cma.j.issn.0254-6450.2017.01.020 28100387

[20] Agzarian J, Visscher SL, Knight AW, Allen MS, Cassivi SD, Nichols FC, Shen KR, Wigle D, Blackmon SH (2019). The cost burden of clinically significant esophageal anastomotic leaks-a steep price to pay. J Thorac Cardiovasc Surg.

[21] Baltin C, Kron F, Urbanski A, Zander T, Kron A, Berlth F, Kleinert R, Hallek M, Hoelscher AH, Chon SH (2019). The economic burden of endoscopic treatment for anastomotic leaks following oncological Ivor Lewis esophagectomy. PLoS One.

[22] Ahrens M, Schulte T, Egberts J, Schafmayer C, Hampe J, Fritscher-Ravens A, Broering DC, Schniewind B (2010). Drainage of esophageal leakage using endoscopic vacuum therapy: a prospective pilot study. Endoscopy.

